# The Influence of Geographic Region on Hip and Knee Arthroplasty Literature From 1988 to 2018

**DOI:** 10.5435/JAAOSGlobal-D-20-00260

**Published:** 2021-06-10

**Authors:** SaTia T. Sinclair, Ahmed K. Emara, Melissa N. Orr, Alison K. Klika, Nicolas S. Piuzzi

**Affiliations:** From the Cleveland Clinic, Department of Orthopedic Surgery, Cleveland, OH.

## Abstract

Total joint arthroplasty constitutes a major focus of publications within orthopaedics. Because research expands and investigators from around the world contribute, it is important to understand the dynamics of publication.

**Methods:**

PubMed was queried for hip and knee arthroplasty-related articles published between 1988 and 2018 within seven orthopaedic journals. A bibliometric analysis was done. The manuscript region of origin was determined by the affiliated country of the last author and used to examine trends in publication.

**Results:**

A total of 6,160 publications were included. Forty-eight countries from six continents were identified. The quantity of arthroplasty-related publications increased over the study period (n = 246 in 1988 and n = 1,247 in 2018, *P* < 0.01). Articles were primarily published by North America (51.9%), Europe (32.5%), and Asia (12.4%). Clinical trials accounted for 45.6% of all publications. Articles from Asia received fewer citations than those from North America, Europe, and Oceania (*P* < 0.001).

**Discussion:**

The volume of publications was five times greater in 2018 than in 1988, yet international articles constitute a marginal proportion of annual publications. Most of the literature (84.4%) originated from North America and Europe. Balanced publication of international research may favor global communication of findings, increasing the spectrum of available evidence applicable worldwide.

Joint arthroplasty is one of the largest subspecialties within orthopaedics and is a common procedure worldwide.^1,2^ More than 1 million total hip arthroplasties (THAs) are done globally every year.^2^ From 2008 to 2017, THAs grew by approximately 37% in the United Kingdom, New Zealand, Sweden, and South Korea.^2^ Common utilization of total knee arthroplasty (TKA) has also been seen worldwide, with several countries reporting nearly 100,000 procedures annually.^1^ As the burden of THA and TKA is estimated to approach nearly 2 million primary joint replacements by 2030 in the United States alone,^3^ sharing information surrounding improved techniques, new technology, and other clinical factors pertaining to these procedures is vitally important. Previous studies have reported increased international contributions to orthopaedic publications.^4,5^ However, these trends have been examined in relatively limited samples, encompassing articles from a minimal number of orthopaedic journals. In addition, trends in orthopaedic publications outside of the United States, particularly for THA and TKA, have not been fully explored.

Clinical research is an essential resource for innovation, and publication of research findings allows necessary dissemination of information. This communication facilitates the adoption of evidence-based practices.^6^ Research productivity (ie, publications in peer-reviewed journals) is widely regarded as a measure of success in academic disciplines.^4,7,8^ Participation in notable research can generate funding for the institution of the investigator, and subsequent authorship can lead to career advancement and attainment of leadership positions.^9,10,11,12^ There is strong competition for publishing in respected journals, as evidenced by rejection rates as high as 84%.^13^ The literature has shown that up to 24.2% of manuscripts rejected from a high-impact orthopaedic journal remain unpublished within the following 5 years.^14^ The inability to publish can limit the career of an orthopaedic surgeon-scientist and may impede dissemination of information among the orthopaedic community at large. Currently, the dynamics of orthopaedic publication across geographic regions remains unknown.

Therefore, the goals of this study were to examine worldwide geographic trends in arthroplasty-related publications to explore the following: (1) volumes and trends of publications in each geographic region over the last 30 years, (2) types of studies published from each region, and (3) citation frequency (ie, citations per article) within each geographic region.

## Methods

A literature search was conducted within the PubMed and MEDLINE databases for THA and TKA articles published in seven high-impact orthopaedic journals with a focus on basic, clinical, and translational research between January 1988 and December 2018. Journals which were not indexed in PubMed for the entire length of the study period were eliminated from consideration. The selected journals included: *Acta Orthopaedica*, *Bone and Joint Journal*, *Clinical Orthopaedics and Related Research*, *International Orthopaedics*, *Journal of Orthopaedic Research*, *Journal of Bone and Joint Surgery (JBJS)-American Volume*, *and Journal of Arthroplasty*. Articles published within *JBJS*-*British Volume* before 2013 were included within the category of the *Bone and Joint Journal*. The search was done using a combination of the following terms: “Arthroplasty, Replacement, Knee” [Mesh], knee replace, knee arthroplasty, “Arthroplasty, Replacement, Hip” [Mesh], hip replace, and hip arthroplasty. Because of the large volume of publications, sampling of articles was done by including all articles for the entire year beginning with 1988 and continuing every 5 years through 2018. This provided 2 years of comprehensive article sampling within each full decade of study. For each year in between, only articles from the first issue of each journal were included to allow a more feasible analysis of the large sample. Data from the intervening years were included in all statistical analyses with the exception of the reported annual trends, which were analyzed solely using data from every 5 years. The search identified 6,351 THA-related and TKA-related articles.

**Figure 1:**
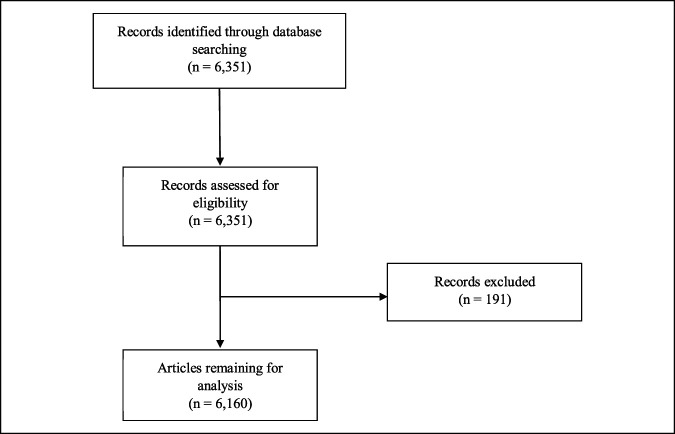
Flowchart showing the selection process of literature evaluated in this study.

A command-line utility from the National Library of Medicine (NLM) known as Entrez Direct, or eDirect, was used to extract the year of publication, journal title, article title, country affiliation of the last author, PubMed ID, number of citations, and PubMed article type for each article. Complete author information was identified through Scopus. The origin of each publication was based on the affiliated country of the last author. Article titles were reviewed for relevance. Only arthroplasty-related articles were included. Publications which did not provide the affiliated country of the last author were excluded (n = 191), leaving 6,160 publications included in this study (Figure 1). A total of 48 countries were identified and separated into six geographic regions based on the continent, including Africa, Asia, Europe, North America, Oceania, and South America. A comprehensive list of the countries within each continent may be found in Supplemental Digital Content (Table S1, http://links.lww.com/JG9/A144).

For each article, the study design was determined by the PubMed article type. Article types which did not reflect the design of the study (eg, research support) were excluded from the analysis of the origin of article types (n = 3,721 excluded), leaving 1,520 available publication types. PubMed article types were classified within eight distinct study design categories, including case report, clinical trial, equivalence trial, evaluation study, meta-analysis, observational study, systematic review, and validation study. The clinical trial category encompassed the controlled clinical trial, randomized controlled trial, and clinical trial PubMed article types. The definitions of all study designs were consistent with those of the respective PubMed article types provided by the NLM. Country affiliation was used to examine quantitative trends in publications within the identified regions. A pairwise Wilcoxon rank sum test with Bonferroni correction was used to compare the number of citations per article between continents. Available PubMed article types were used to evaluate the proportion of study designs produced in each region.

## Results

**Figure 2:**
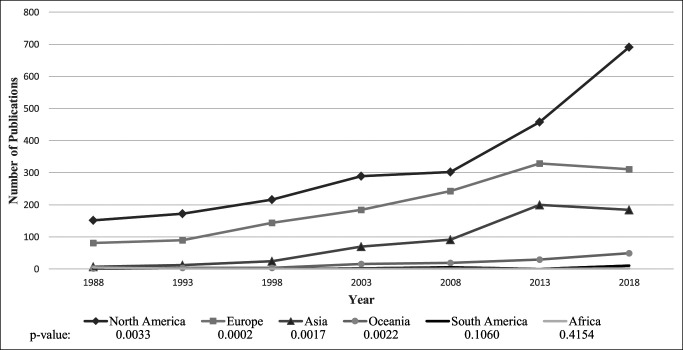
Graph showing the annual number of publications in each region plotted over time (1988 to 2018).

**Figure 3:**
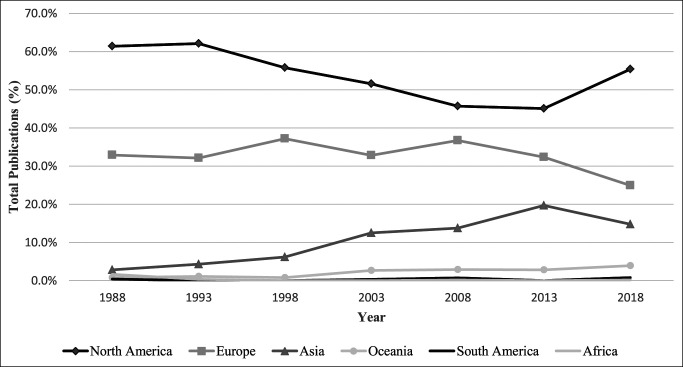
Graph showing the percentage of total annual publications contributed by each region plotted over time (1988 to 2018).

Overall, the total number of arthroplasty-related publications within the seven orthopaedic journals increased by 407% from 1988 to 2018 (n = 246 in 1988 and n = 1,247 in 2018) (Figure 2). North America produced the largest quantity of publications over the 30-year study period (n = 3,199), accounting for 51.9% of total publications (Table 1). Europe (n = 2000) and Asia (n = 762) contributed 32.5% and 12.4% of total publications, respectively. By contrast, Oceania (n = 168, 2.7%), South America (n = 21, 0.3%), and Africa (n = 10, 0.2%) generated only a small portion of published articles. The quantity of publications produced by North America in 2018 (n = 691) was more than double the number of publications produced within the continent in 2008 (n = 302; *P* = 0.003) (Figure 2). Both Europe (n = 89 in 1993, n = 329 in 2013; *P* < 0.001) and Asia (n = 12 in 1993 and n = 200 in 2013; *P* = 0.002) had a substantial increase in arthroplasty-related publications from 1993 to 2013. However, the number of publications from these continents decreased by 5.5% and 8.0%, respectively, from 2013 to 2018. By contrast, the number of publications from North America increased by 51% (n = 458 in 2013, n = 691 in 2018; *P* = 0.003) within the same time frame. North America and Europe consistently made the largest contributions to the total amount of arthroplasty-related articles each year (Figure 3). From 1993 to 2013, the proportion of total annual articles contributed by North America decreased from 62.1% to 45.1% (−17%), whereas the proportion of total annual articles contributed by Asia simultaneously increased from 4.3% to 19.7% (+15.4%). On average, publications from Oceania, Africa, and South America constituted 2.3%, 0.21%, and 0.20% of total annual publications during the entire study period, respectively. The United States (n = 2900), the United Kingdom (n = 665), Japan (n = 260), Australia (n = 132), Brazil (n = 7), and South Africa (n = 7) generated the most publications within their respective continent (Table 1). The contribution of publications made by each of the 48 included countries is provided in Supplemental Digital Content (Table S2, http://links.lww.com/JG9/A145).

**Table 1:** Total Publications by Region (1988-2018)

Region	Country-Specific Number of Publications (%)	Total Number of Publications (%)
North America		3,199 (51.9)
The United States	2,900 (47.1)	
Canada	296 (4.8)	
Mexico	3 (0.05)	
Europe		2,000 (32.5)
The United Kingdom	665 (10.8)	
Sweden	224 (3.6)	
Germany	181 (2.9)	
Asia		762 (12.4)
Japan	260 (4.2)	
South Korea	183 (3.0)	
China	151 (2.5)	
Oceania		168 (2.7)
Australia	132 (2.1)	
New Zealand	36 (0.6)	
South America		21 (0.3)
Brazil	7 (0.1)	
Argentina	7 (0.1)	
Colombia	5 (0.08)	
Africa		10 (0.2)
South Africa	7 (0.1)	
Egypt	3 (0.05)	

The top three publishing countries for each region are displayed as applicable.

**Figure 4:**
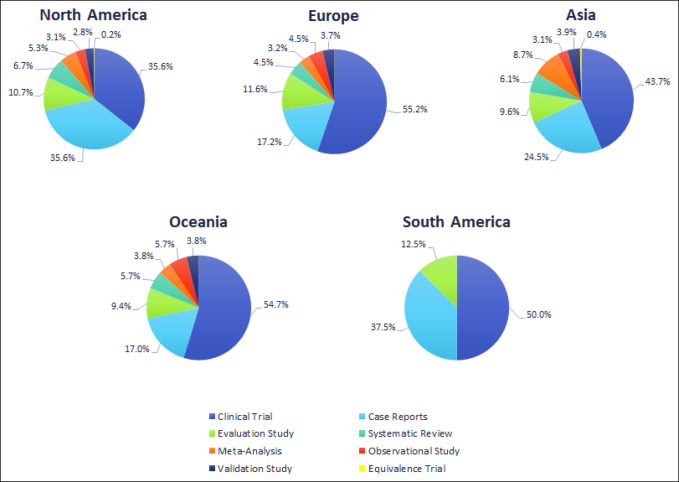
Pie chart showing the distribution of study designs within each region is displayed (1988 to 2018). Clinical trial includes controlled clinical trial, randomized controlled trial, and clinical trial PubMed article types. Definitions of these study designs are consistent with those of the PubMed article types provided by the National Library of Medicine.

Clinical trials, case reports, and evaluation studies accounted for 45.6% (n = 693), 25.8% (n = 392), and 10.9% (n = 165) of all study designs, respectively. North America published clinical trials and case reports (n = 217, 35.6% for both) in equal volumes (Figure 4). Overall, 52.3% of studies published outside of North America were clinical trials (Europe: n = 343, 37.7%; Asia: n = 100, 11%; Oceania: n = 29, 3.2%; and South America: n = 4, 0.4%). None of the 10 arthroplasty-related articles generated by Africa possessed a PubMed article type which corresponded to study design.

**Figure 5:**
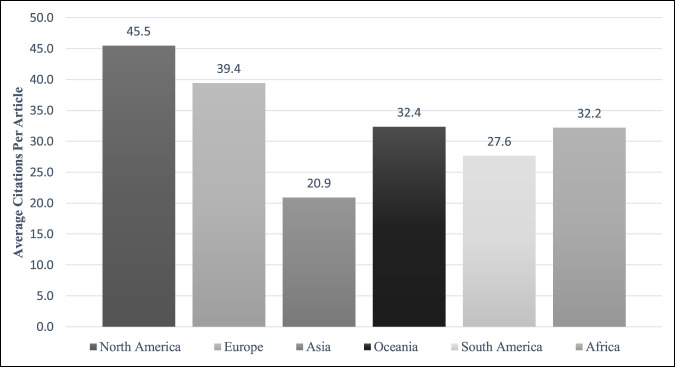
A bar graph showing the average number of times the articles from each region have been cited.

The mean number of citations per article between 1988 and 2018 ranged from 20.9 (±26.9) for publications from Asia to 45.5 (±70.1) for publications from North America (Figure 5). Publications from Asia received significantly less citations than those originating from North America, Europe, and Oceania (*P* < 0.001). Comparisons between all other regions revealed no statistically significant differences in citations across continents (*P* > 0.05) (Table 2).

**Table 2:** Pairwise Wilcoxon Rank Sum Test for the Mean Number of Citations (1988 to 2018)

Country (Mean Citations)	Comparison	*P*
	Africa–Asia	1
	Africa–Europe	1
North America (45.5)	Africa–North America	1
	Africa–Oceania	1
Europe (39.4)	Africa–South America	1
	Asia–Europe	1.10E-20
Asia (20.9)	Asia–North America	6.12E-31
	Asia–Oceania	0.012
Oceania (32.4)	Asia–South America	1
	Europe–North America	0.052
South America (27.6)	Europe–Oceania	1
	Europe–South America	1
Africa (32.2)	North America–Oceania	0.183
	North America–South America	1
	Oceania–South America	1

Articles within four of the selected orthopaedic journals primarily originated from North America, whereas the remaining three journals were largely comprised of publications from Europe (Table 3).

**Table 3:** Source of Publications for Each Journal (1988-2018)

*Acta Orthopaedica* (n = 472)				
	North America	5.9%	Oceania	2.8%
	Europe	84.3%	South America	0.2%
	Asia	6.8%	Africa	0.0%
*Bone and Joint Journal*^a^ (n = 758)				
	North America	23.2%	Oceania	4.0%
	Europe	63.6%	South America	0.3%
	Asia	8.8%	Africa	0.1%
*Clinical Orthopaedics and Related Research* (n = 1218)				
	North America	70.2%	Oceania	2.2%
	Europe	20.1%	South America	0.2%
	Asia	7.1%	Africa	0.2%
*International Orthopaedics* (n = 440)				
	North America	9.5%	Oceania	1.1%
	Europe	54.8%	South America	1.4%
	Asia	32.7%	Africa	0.5%
*Journal of Arthroplasty* (n = 2409)				
	North America	61.6%	Oceania	3.1%
	Europe	19.1%	South America	0.4%
	Asia	15.6%	Africa	0.2%
*Journal of Bone and Joint Surgery-American Volume* (n = 711)				
	North America	76.1%	Oceania	2.1%
	Europe	15.9%	South America	0.0%
	Asia	5.8%	Africa	0.1%
*Journal of Orthopaedic Research* (n = 152)				
	North America	48.0%	Oceania	2.6%
	Europe	39.5%	South America	0.0%
	Asia	9.9%	Africa	0.0%

a Includes *Journal of Bone and Joint Surgery-British Volume*.

## Discussion

Publication of scholarly work is critically important for both the career of the orthopaedic surgeon and the global distribution of research findings, which may lead to improved clinical outcomes for orthopaedic patients.^6,11^ Although there has been increased internationalization of orthopaedic literature, there remains a paucity of global representation in arthroplasty literature.^4,15^ Therefore, we sought to examine longitudinal trends in arthroplasty publications from geographic regions across the world relating to volume, study design, and citations over the past 30 years.

This study was strengthened by using a larger sample size than previous literature, including fewer orthopaedic journals^4,15,16^; however, limitations were still present. This study only examined journals printed in English that were indexed in PubMed for the entire length of the study period. This study focused on quantitative data and did not evaluate the quality of the publications included (eg, level of evidence and sources of citations). For select years, only articles from the first issue of each journal were included as previously stated within the methods. We recognize that this strategy underestimated the number of publications during intervening years; however, this method of inclusion was used consistently across all intervening years for all journals. It should be noted that the reported annual data in this study were derived from years in which articles from the entire year were included (ie, every fifth year). We also acknowledge that the current results were obtained without respect to the number of surgeons within each region, although less published regions, such as China, reported the presence of over 40,000 orthopaedic surgeons who regularly do hip and knee arthroplasty.^17^ Finally, the study designs of each publication were determined by the listed PubMed article types. This information is not always included for each study in PubMed, and it is possible for one publication to have more than one PubMed article type. However, this study yielded 1,520 article types, which provided a large sample for analysis. The consistent use of the NLM definitions allowed uniformity across the sample.

The results of this study showed that the volume of THA and TKA publications was five times greater in 2018 than in 1988, yet international articles continue to form a marginal proportion of total annual arthroplasty-related publications. A large majority (84.4%) of arthroplasty literature between 1988 and 2018 originated from North America and Europe. Noteworthy is that journals such as *Clinical Orthopaedics and Related Research* and *Bone and Joint Journal* produce publications from the proceedings of meetings which occur primarily in North America. Such publications contribute to the overall volume of literature originating in North America, potentially skewing the observed results.

Publications from Oceania, South America, and Africa consistently comprised less than 10% of total annual publications when combined, yet articles from these regions were cited just as often as those from North America and Europe. Composition of the study design was also shown to be similar in North America, Europe, Asia, and Oceania. The observed similarities in citations and study designs across regions indicate that articles from less frequently published regions shared comparable relevance. Potential differences in manuscript submission rates across regions should be considered because this may affect the publication rate within the respective countries. Additional investigation is needed to ascertain the rate of manuscript submission within each geographic region. In addition, submission of manuscripts to journals with less stringent requirements for publication could also contribute to a lower volume of published material within high-impact journals.

The findings of this study are consistent with those of previous work conducted by Camp and Escott,^4^ which noted trends in general orthopaedic publications. This study evaluated research articles published within *JBJS-American Volume* and *JBJS-British Volume* and reported that 59.5% and 33.9% of the articles originated from North America (ie, the United States and Canada) and the United Kingdom and Ireland, respectively.^4^ Similar to our study, the number of publications originating from North America steadily increased, yet the proportion of articles from this region decreased to 40.4% by 2009.^4^ In addition, Camp and Escott^4^ observed an increase in the proportion of publications from Asia (1.8% in 1979 to 10.5% in 2009). Despite these similarities, not all the results presented by Camp and Escott^4^ were uniform with this study. Although the number of articles originating from the United Kingdom and Ireland increased over the study period, Camp and Escott^4^ reported that the proportion of publications contributed by this region was 20.6% in 2009, nearly half of the proportion contributed by this region in our study at that time. Different classification of regions, inclusion of a greater number of orthopaedic journals, and a larger sample size within this study likely accounted for this discrepancy.

Additional studies offer insight into the observed trends.^15,16^ Okike et al.^15^ evaluated manuscripts submitted to *JBJS* and reported that submissions from the United States or Canada had a rate of acceptance for publication of 28.1%, whereas manuscripts submitted from other countries had an acceptance rate of 14.2% (odds ratio = 0.51, 95% confidence interval: 0.28 to 0.92). Similarly, Lynch et al.^16^ reported an increased likelihood of acceptance for publication in *JBJS-American Volume* if manuscripts originated from the United States (*P* = 0.020). At the time of this study, Lynch et al revealed that 39% of manuscripts from the United States were accepted for publication in comparison with 22% of manuscripts from foreign countries.^16^ Investigators stated that these findings were observed, despite the lack of differences in quality of research studies. Lynch et al suggested that peer reviewers within the United States, although blinded to the manuscript region of origin, may have been likely to recognize study designs and language that they found more familiar.^16^ Although this is not the case with every journal, when true, this bias may cause such studies to receive better reviews.^16^ Journals may benefit from incorporating a diverse group of peer reviewers within the publication process because a lack of diversity among reviewers could also introduce cultural bias, rendering certain topics less likely to be accepted for publication because of lack of interest and variations in practice between regions. Other potential factors influencing publication, such as concealing or delaying results, insufficient resources, and inadequate training, have also been suggested.^8,16,18,19,20,21,22,23,24,25^ When present, the requirement of paying a submission fee could also serve as a deterrent for publication for investigators in low-income countries.

Global research provides a means to develop a more comprehensive and objective perspective of patient care. Failure to publish international research inhibits global communication of findings, leaving a narrow spectrum of available evidence that may not be applicable to patient populations worldwide. Thus, both increased research productivity and receptivity are needed within arthroplasty research. Future studies may examine whether differences exist in the aforementioned trends between THA-related and TKA-related publications. The incidence of arthroplasty surgery and the rate of manuscript submission across geographic regions are also avenues for future investigation.

## Supplementary Material

SUPPLEMENTARY MATERIAL
