# Supplementary material for: The Influence of Geographic Region on Hip and Knee Arthroplasty Literature From 1988 to 2018
Source: J Am Acad Orthop Surg Glob Res Rev. 2021 Jun 10;5(6):e20.00260. doi: 10.5435/JAAOSGlobal-D-20-00260 (PMC8196095; doi:10.5435/JAAOSGlobal-D-20-00260)
Supplement: SUPPLEMENTARY MATERIAL [file jagrr-5-e20.00260-s001.docx]

**Table S1** Countries Included within Each Continent.

| **Africa** | **Europe** | **North America** |
| --- | --- | --- |
| Egypt | Austria | Canada |
| South Africa | Belgium | Mexico |
|  | Bulgaria | United States |
| **Asia** | Croatia |  |
| China | Czech Republic |  |
| India | Denmark | **Oceania** |
| Iran | Finland | Australia |
| Israel | France | New Zealand |
| Japan | Germany |  |
| Lebanon | Greece |  |
| Malaysia | Ireland |  |
| Saudi Arabia | Italy | **South America** |
| Singapore | Netherlands | Argentina |
| South Korea | Norway | Brazil |
| Taiwan | Poland | Chile |
| Thailand | Portugal | Colombia |
| Turkey | Romania |  |
| United Arab Emirates | Serbia |  |
|  | Slovenia |  |
|  | Spain |  |
|  | Sweden |  |
|  | Switzerland |  |
|  | United Kingdom |  |
