# Supplementary material for: The Influence of Geographic Region on Hip and Knee Arthroplasty Literature From 1988 to 2018
Source: J Am Acad Orthop Surg Glob Res Rev. 2021 Jun 10;5(6):e20.00260. doi: 10.5435/JAAOSGlobal-D-20-00260 (PMC8196095; doi:10.5435/JAAOSGlobal-D-20-00260)
Supplement: SUPPLEMENTARY MATERIAL [file jagrr-5-e20.00260-s002.docx]

**Table S2** Total Publications and Percentage of Total Publications by Country (1988 – 2018).

| **Country** | **Total Publication Count, By Country** | **Percentage Total Publication Count, By Country** |
| --- | --- | --- |
| United States | 2900 | 47.1% |
| United Kingdom | 665 | 10.8% |
| Canada | 296 | 4.8% |
| Japan | 260 | 4.2% |
| Sweden | 224 | 3.6% |
| South Korea | 183 | 3.0% |
| Germany | 181 | 2.9% |
| Netherlands | 152 | 2.5% |
| China | 151 | 2.5% |
| France | 146 | 2.4% |
| Australia | 132 | 2.1% |
| Denmark | 106 | 1.7% |
| Finland | 80 | 1.3% |
| Italy | 74 | 1.2% |
| Switzerland | 69 | 1.1% |
| Spain | 69 | 1.1% |
| Norway | 68 | 1.1% |
| Austria | 57 | 0.9% |
| India | 44 | 0.7% |
| Taiwan | 42 | 0.7% |
| New Zealand | 36 | 0.6% |
| Belgium | 35 | 0.6% |
| Greece | 29 | 0.5% |
| Singapore | 28 | 0.5% |
| Ireland | 22 | 0.4% |
| Turkey | 17 | 0.3% |
| Thailand | 15 | 0.2% |
| Israel | 10 | 0.2% |
| South Africa | 7 | 0.1% |
| Brazil | 7 | 0.1% |
| Argentina | 7 | 0.1% |
| Slovenia | 6 | 0.1% |
| Czech Republic | 6 | 0.1% |
| Colombia | 5 | 0.08% |
| United Arab Emirates | 4 | 0.06% |
| Poland | 4 | 0.06% |
| Saudi Arabia | 3 | 0.05% |
| Mexico | 3 | 0.05% |
| Iran | 3 | 0.05% |
| Egypt | 3 | 0.05% |
| Croatia | 3 | 0.05% |
| Chile | 2 | 0.03% |
| Serbia | 1 | 0.02% |
| Romania | 1 | 0.02% |
| Portugal | 1 | 0.02% |
| Malaysia | 1 | 0.02% |
| Lebanon | 1 | 0.02% |
| Bulgaria | 1 | 0.02% |
